# EAT-Lancet Diet Components Acquisition According to Food Insecurity and Poverty Status in Brazil: An Analysis of National Household Budget Survey 2017–2018

**DOI:** 10.3390/ijerph22050808

**Published:** 2025-05-21

**Authors:** Eduardo De Carli, Mariana Alves Ferreira, Lucas de Almeida Moura, Valéria Troncoso Baltar, Dirce Maria Lobo Marchioni

**Affiliations:** 1 Department of Nutrition, School of Public Health, University of São Paulo, São Paulo 01246-904, SP, Brazil; edecarli@usp.br (E.D.C.); ferreira.almariana@usp.br (M.A.F.); lucasdemoura@usp.br (L.d.A.M.); 2 Department of Epidemiology and Biostatistics, Institute of Collective Health, Fluminense Federal University, Niterói 24030-215, RJ, Brazil; vtbaltar@id.uff.br

**Keywords:** hungry, income, expenditure, sustainability, healthy diet

## Abstract

The EAT-Lancet diet outlines target consumption for specific food components but overlooks accessibility and cost issues, which may hinder adherence among vulnerable populations. This study examines the acquisition profile of EAT-Lancet diet components by food security and poverty status, using data from 57,920 households in the 2017–2018 Brazilian Household Budget Survey. Poverty and food insecurity were defined according to the World Bank per capita income cutoffs and the Brazilian Food Insecurity Scale, respectively. Food acquisition was classified into 15 EAT-Lancet diet components and expressed as per capita daily averages (g, % of total available energy, and % of food expenditure), by food security and poverty strata. Brazilian households were 37.9% food-insecure and 12% poor. Compared to more privileged counterparts, these households prioritized the acquisition of staples like refined cereals and legumes over most EAT-Lancet diet adequacy components, such as fruits, vegetables, whole grains, nuts, and peanuts. While lower energy shares from moderation components were only slightly evident for red meat and dairy among food-insecure households, pronounced reductions in added sugars and vegetable oils were seen among the poor. These findings suggest that public policies should synergically address particularities of different deprivation contexts to promote sustainable diets in Brazil.

## 1. Introduction

In 2019, the EAT-Lancet Commission proposed a global reference framework—the Planetary Health Diet—to establish scientific targets for sustainable food systems. This framework defines optimal consumption ranges for various food groups to simultaneously promote human and environmental health [1]. Using scenario modeling, the Commission assessed the diet’s alignment with greenhouse gas emission reductions and global climate goals, concluding that achieving such a dietary shift by 2050 would require significant changes in global eating patterns. These include a substantial reduction in starchy foods and red meat consumption, coupled with increased intake of legumes, whole grains, and nuts [1]. However, the adoption of healthy and sustainable diets depends critically on financial accessibility and food security, particularly among populations in low-income countries [2].

Food security is a multidimensional concept supported by six fundamental pillars: availability, access, utilization, stability, agency, and sustainability [3]. Among these, access—understood as the physical and economic capacity to obtain food—is the primary focus of both global monitoring efforts and public policy interventions on food security [3,4]. Given that economic inequality and high food prices are major barriers to accessing healthy diets, many public programs seek to increase household income—i.e., reduce poverty—as a strategy to decrease food insecurity [3,4]. However, according to the Food and Agriculture Organization of the United Nations (FAO), while there is strong evidence of overlap between food insecurity and poverty at the household level in countries of sub-Saharan Africa and Latin America, it is also recognized that limited access to food may not necessarily stem solely from financial constraints. Conversely, other households, even with resources limited to subsistence, may still achieve food security [4].

In Brazil, food security has deteriorated significantly over the past decade. Severe household food insecurity—where all residents experience hunger—rose sharply from 4.6% in 2018 to 15.5% in 2022, during the second year of the COVID-19 pandemic [5,6]. Concurrently, 23.5% of Brazilians lived in poor households, according to the World Bank’s international monetary poverty threshold for upper-middle income countries [7]. It is also estimated that 57% of the Brazilian adult population are overweight [8], while only 1% consume a diet classified as low risk for nutritional deficiencies and noncommunicable diseases [9]. These data underline the complex relationship between food systems and health outcomes, which, coupled with climate change, characterize what is defined as a global syndemic [10]. This multifaceted crisis underscores the barriers to adopting healthy and sustainable diets in the country.

In fact, although the concept of healthy and sustainable eating is included in Brazil’s national dietary guidelines [11], adherence to the EAT-Lancet diet remains limited. Studies using the Planetary Health Diet Index (PHDI) and food consumption data from the most recent Brazilian Household Budget Survey (HBS 2017–2018) showed that individuals over 10 years old reached, on average, only one-third of the maximum PHDI score [12]. Moreover, there is evidence of even lower EAT-Lancet diet adherence among those in food insecurity, even not necessarily in a low-income situation [13]. On the other hand, data at the household level suggest that the average cost to achieve EAT-Lancet recommendations exceeds that of the typical diet consumed by most Brazilians [14]. In this sense, trends indicate that this cost can be reduced by lowering the acquisition of moderation/limited components, such as meats and dairy products, while increasing the intake of adequacy/emphasized components, such as fruits, vegetables, whole grains, nuts, and peanuts, tends to raise costs, particularly among low-income households [14,15]. Jointly, these findings reinforce international concerns about the economic feasibility of healthy and sustainable dietary models, such as the EAT-Lancet diet, given their financial inaccessibility for large segments of the global population, especially in middle- and low-income countries [2,16]. Furthermore, they suggest possible divergences in food acquisition and consumption patterns among Brazilians from food-insecure versus low-income households.

In this context, a better understanding of the relationship between poverty, food insecurity, food access, availability, and consumption is crucial for policymakers. Specifically, examining household food acquisition behaviors could shed light on how dietary sustainability is influenced by different deprivation contexts, while also underscoring nutritional education actions among vulnerable populations. The Household Budget Survey (HBS) 2017–2018 offers a unique opportunity for such analysis, as it was the first national survey to integrate data on household income, food security, food acquisition, and food consumption. Therefore, this study aims to describe the composition of household food acquisition in Brazil, considering food security and poverty status, in alignment with the components of the EAT-Lancet reference diet.

## 2. Materials and Methods

### 2.1. Household Budget Survey 2017–2018

This is a cross-sectional study utilizing data from the Household Budget Survey (HBS) 2017–2018, carried out by the Brazilian Institute of Geography and Statistics (IBGE) [17]. The HBS investigated structured aspects of living conditions of Brazilians, obtaining detailed information regarding incomes, food security, and food acquisition [6,17,18]. A two-stage cluster sampling design was employed, based on geographical and socioeconomic stratifications derived from the 2010 Demographic Census and the IBGE’s Integrated Household Survey System. Briefly, IBGE’s divides the country into census sectors according to regions, urban/rural status, and household incomes. In the first stage, within defined strata of sectors with geographic and socioeconomic homogeneity, 5504 primary sampling units (PSUs—i.e., sectors or aggregates of sectors) were randomly selected proportionally to the number of households within census sectors. In the second stage, households were randomly selected within each PSU. The final sample comprised 57,920 households, aggregated into 575 strata (3 to 73 PSU per stratum). Each household stratum was followed over a 12-month period, from July 2017 to July 2018, with interviews uniformly distributed throughout the year to capture the seasonality of food consumption and costs [17]. In each household, a set of socioeconomic and demographic data was collected, including information on the Brazilian region (North, Northeast, Southeast, South, and Central-West) and area of residence (urban or rural), as well as the age, gender, income, and years of schooling of the household head and residents [6,17,18]. Interviews were conducted using validated national and international instruments and protocols, following standardized operational plans and using electronic data collection tools to ensure consistency and quality [6,17,18].

As HBS uses a complex sample, post-stratification sampling weights were applied. These IBGE’s weights were adjusted for variations in sampling probabilities across strata and were calibrated using demographic and socioeconomic variables such as age, sex, and region, seeking to align the sample with known population totals based on the 2010 Census projection to 2018, thus ensuring that the weighted sample accurately reflects the Brazilian population [17]. Additional details on sample design, questionnaires, data quality control, and variables imputation are available in official publications [6,17,18]. All microdata is freely accessible via the IBGE’s website [19].

### 2.2. Food Insecurity and Poverty Status

Food security status was assessed using the validated Brazilian Food Insecurity Scale (EBIA, acronym in Portuguese) [20]. EBIA comprises 14 yes/no questions, including 8 items that apply to households with only adults (≥19 years) and 6 items that apply to households with children/adolescents (<18 years) [20]. The household head completed the questionnaire, which covered the food security experience during the preceding 90 days [6]. Based on standard cut-off points, households were categorized as follows: (1) food security (regular and permanent access to sufficient, quality food); (2) mild food insecurity (concern or uncertainty about future food access); (3) moderate food insecurity (disrupted eating patterns due to food scarcity and/or reduced food intake among adults); and (4) severe food insecurity (reduced intake among all household residents, including children, indicating experienced hunger) [6,20].

To classify households not covering basic nutritional requirements (food poverty) as well as non-food necessities (total poverty), we adopted per capita household disposable income thresholds specifically calculated by the World Bank Group based on the HBS 2017–2018 data [21]. For that, household monetary and non-monetary incomes were summated, and taxes were discounted [22]. Daily disposable incomes were then divided by the total number of residents and each household was classified as follows: (1) above the poverty line (≥BRL 15.0 or USD 4.7); (2) below total poverty line (<BRL 15.0 or USD 4.7); and (3) below food poverty line (<BRL 8.5 or USD 2.7) [21].

For this study, food security and poverty status were dichotomized (without vs. with food insecurity; and above vs. below the total poverty line) or stratified into three levels (without vs. mild and moderate vs. severe food insecurity; and above the total poverty line vs. below the total and above the food poverty line vs. below the food poverty line), respectively.

### 2.3. EAT-Lancet Diet Components Acquisition

Household food, beverages, and non-food items acquisitions were recorded over a one-week period, either by residents or by an IBGE interviewer, detailing descriptions of the products, places of purchase, expenditure type (monetary or non-monetary), unit price (BRL), and amounts (g or mL). After excluding non-food, alcoholic beverages, and nonspecific or aggregated items, 4194 different food entries were identified. Initially, these items were aggregated into 760 groups, according to their conceptual and nutritional similarities (e.g., types of beans). Of these, 508 groups were directly matched to single codes from the Brazilian Food Composition Table (TBCA-USP) [23], which aided conversion to energy values. The remaining groups, comprising different mixed preparations (e.g., sandwiches, ready-to-eat meals, fried potatoes, etc.) or industrialized products (e.g., cookies, soft drinks, savory snacks, etc.), were disaggregated into their main ingredients. This was achieved through nutrient fractioning (e.g., fats content in a savory chip was assumed as the fraction of the vegetable oil group’s contribution to that food) or standard recipes disaggregation, based on TBCA-USP data [24], as previously detailed elsewhere [25]. Finally, foods and ingredients were categorized into 15 EAT-Lancet diet components, following adaptations proposed by Cacau et al. (2021) for the Planetary Health Diet Index (PHDI) [25], as summarized in Table 1.

Prior to aggregating weekly amounts (g) and energy (kcal) from individual food components acquired by each household, correction factors to inedible portions (e.g., peels, shavings, and pits) were applied, when appropriate [26]. In turn, each expenditure (BRL) on a mixed preparation or industrialized products was firstly associated with a single EAT-Lancet component, according to predominant ingredients in the item, to compute household weekly totals. That approach, despite deviating from full decomposition of mixed-foods’ amount and energy contents, was required for items covering less than one-sixth of total households’ food expenditures, thus, not greatly affecting the overall data interpretation.

To facilitate the interpretation of findings, we assumed the original classification of EAT-Lancet components proposed in the Planetary Health Diet Index (PHDI) (Table 1), grouping them dichotomously as either adequacy components (nuts and peanuts, legumes, fruits, vegetables, and whole cereals) or moderation components (red meat, chicken, animal fats, added sugars, eggs, dairy products, fish and seafood, tubers and potatoes, vegetable oils, and refined cereals). This approach considered that, in Brazil, both moderation and optimum components, excepting vegetable oils and fish and seafoods, have shown average population intakes above EAT-Lancet reference diet recommendations, indicating inadequacies due to excess, as opposed to adequacy components [12,25].

### 2.4. Statistical Analysis

Given that the HBS captured food acquisition data over a short reference period (one week), sampling strata as a whole (n = 575), as well as split by household food security status (n = 1148 and 1645) or poverty status (n = 1117 and 1516), were used as study units. The average number of households studied within these sets of study units ranged from 38.2 to 100.7. After weekly totals were converted to express daily values, per capita EAT-Lancet diet components acquisition averages were estimated by summing their expenditures (BRL), amounts (g) and energy availability (kcal) and dividing these by the total number of individuals within each sampling stratum. Additionally, the share of each component in total food expenditure (% of food expenditure/capita/day) and total food energy (% of available energy/capita/day) were calculated. Similarly, household strata were characterized using prevalence indicators of rural/urban area, Brazilian regions, as well as gender (female/male), age (<18/≥18 years), and self-reported skin color (white/non-white) of the household head and residents. The average of the residents per household, per capita disposable income and the age and years of schooling (for individuals aged ≥18 years) of both the household head and residents were also reported.

Standard errors and 95% confidence intervals were calculated considering sampling weights and the complex sampling design of the surveys. The comparison between 95% CI values was used to identify significant differences. The absence of overlap between the intervals was assumed as a significant difference, considering the level of significance of 5%. Currency exchange of income and expenditures values assumed the reference date of 15 January 2018 (BRL 1 = USD 3.19) [27]. All analyses were performed using the STATA statistical package version 17·0 (Stata Corp LLC, College Station, TX, USA).

## 3. Results

Table 2 presents selected characteristics of sampling strata. Less than one-quarter of households were situated in the rural area, and nearly 70% concentrated in Southeast (41.6%) and Northeast (27.7%) Brazilian regions (Table 2). Daily per capita income was USD 19.3. The households were composed of an average of 3.0 residents, aged nearly 35 years, with one-quarter aged <18 years. Approximately 55% were headed by a non-white person, and 51.3% by a woman. Among adult residents, the average number of years of schooling was about 9 (Table 2).

Almost 40% of households were food-insecure (37.9%), including 4.9% in the most severe degree. Meanwhile, 12% had an average daily per capita income below the poverty line, with 4% below the food poverty line (Table 2). As presented in Table 3, households in both vulnerable situations had on average a larger number of residents, with lower age, more frequently self-reported as non-white, and with fewer number of years of schooling. However, households below the poverty line exhibited more pronounced differences, especially in household size and age structure, averaging about one more resident per household and about 10 fewer years of age for both heads and residents in relation to households above the poverty line (Table 3). Households in food insecurity, but not below the poverty line, were more frequently headed by a woman (Table 3), especially in the most severe degree (Table S1). A lower number of years of schooling for both heads and adult residents, and a higher proportion of non-white residents also occurred in households with severe food insecurity in relation to those with higher degrees of food security (Table S1). Particularly, in households below the food poverty line, a slightly lower frequency of women residents was found (Table S1). The difference in daily per capita incomes was about 50% between households with and without food security and 80% between those above and below the poverty line (Table 2), with averages as low as USD 10.7 and USD 1.6 for those experiencing severe degrees of food insecurity and food poverty, respectively (Table S1).

Table 4 presents data on average daily per capita expenditure, available total amount and energy contribution of food acquisitions by households in situations of food insecurity and poverty. The per capita food expenditures of such households were, on average, 33.3% and 42.9% lower than those in food security and above the poverty line, amounting to approximately USD 1.0 and 0.8 daily, respectively. Regardless of household socioeconomic status, red meat and refined cereals accounted for around 45% of total food expenditure. However, higher relative expenditure on refined cereals and legumes were found in both vulnerable situations (Table 4). In turn, relative to the respective most privileged counterparts, lower expenditure on red meat occurred in households below the poverty line (Table 4), and in those in the severe degree of food insecurity (Table S2). Food-insecure households also showed higher expenditure on chicken and vegetable oils, while lower on dairy products in comparison to food-secure ones (Table 4). In both vulnerable situations, lower relative expenditures on nuts, fruits, vegetables, whole grains, and animal fats were found (Table 4).

Differences in the daily per capita total available amounts of food between households with and without food insecurity and between those below and above the poverty line were 22.2% and 35.2%, respectively. Such differences were driven by lower per capita availability of most EAT-Lancet components in both vulnerable situations, except for the legumes, and in food-insecure households, also for the refined cereals (Table 4).

Averages of about 180 and 385 fewer daily per capita available calories were found between households with and without food insecurity and between those below and above the poverty line, representing relative differences of 13.6% and 29.6%, respectively (Table 4). Refined cereals, added sugars, and vegetable oils accounted for >60% of the total energy available in all household status studied (Table 4). However, vulnerable households, in comparison to their respective counterparts, exhibited higher shares of energy from refined cereals in association with lower shares from fruits, vegetables, whole cereals, nuts and peanuts, and animal fats (Table 4). In addition, food-insecure households showed slightly lower energy share from red meats and dairy products (Table 4). Particularly, lower energy share from added sugars and vegetable oils was found in households below the poverty line relative to those in all other studied socioeconomic situations (Table 4).

## 4. Discussion

The present study evaluated the composition of food acquisition by Brazilian households according to food security and poverty status, based on components of the EAT-Lancet reference diet. Our findings indicate that, despite differences of approximately 30% and 50% in total per capita food expenditures, households experiencing food insecurity and those below the poverty line exhibited per capita energy availability levels close to 90% and 70% of those food-secure and above the poverty line, respectively. In these contexts, a greater share of the food budget within households in vulnerable situations was dedicated to energy-contributing staple items of the Brazilian diet, such as refined cereals and legumes, at the expense of most EAT-Lancet adequacy components, such as fruits, vegetables, whole grains, and nuts and peanuts. Moreover, according to our findings, compensatory reductions in the food expenditure with high-cost items, such as animal-sourced foods, occurred for animal fats while were more evident for dairy products among food-insecure households and for red meat among those below the poverty line. However, by allocating a greater share of total per capita available energy with refined cereals and a lower share with most adequacy components, except legumes, imbalances in relation to EAT-Lancet diet recommendations emerged more evident in vulnerable households, when compared to those in food security and above the poverty line. It is worth noting that apparent inadequacies due to excessive shares of moderation components, such as red meat and added sugars, and insufficient shares of fruits, vegetables, whole grains, legumes, and nuts and peanuts, were observed across all Brazilian household strata, regardless of the studied socioeconomic status.

Between 2017 and 2018, 37.9% of Brazilian households experienced some degree of food insecurity, while 12% were below the poverty line, including approximately 5% and 4% in the most severe situations of hunger and food poverty, respectively. According to our data, among households severely food-insecure and below the food poverty line, approximately 7.5% and 56.2% of the daily per capita disposable income (USD 10.7 and USD 1.6) were allocated to the acquisition of food for home consumption (USD 0.8 and USD 0.9), respectively. Interestingly, in all studied household status, nearly half of total food expenditure was allocated solely to refined cereals and red meat. These results highlight a significant disparity between the current food consumption patterns of Brazilians and the projected budget distribution to meet all EAT-Lancet reference diet targets for Latin America and the Caribbean countries, which limit daily shares of food expenditure on staple starchy foods and animal-sourced foods to 15% and 25%, respectively [2]. Since food for home consumption accounts for more than two-thirds of total food expenditures in the Brazilian population [17], these findings indicate that households experiencing food poverty currently compromise disposable incomes nearly twice the projected for upper-middle-income countries in a scenario with all EAT-Lancet diet recommendations met [2]. National data reinforce the existence of financial barriers to access healthy diets in Brazil, since the current share of food acquisition represents the second-largest component of total expenditures, which, in turn, is 20% and 40% higher for households facing mild and severe degrees of food insecurity relatively those in food security [6], and almost 3 times higher for those in the lowest compared to the highest income class [17]. Additionally, according to a modeling study, achieving full compliance with the EAT-Lancet diet recommendations would require an increase of approximately 9% of current total food expenditures among Brazilian households [14].

In line with our findings, Caldeira et al. (2025) have recently described an inverse correlation between adherence to EAT-Lancet diet recommendations and food costs in Brazil [15]. The authors attributed this association to a dynamic of reducing diet costs by lowering consumption of pricier moderation components, such as meat and dairy products, among low-income households facing financial constraints [15]. Based on our results, slightly lower expenditure shares on dairy products among food-insecure households and on red meat among those below the poverty line were indeed evidenced, despite a higher expenditure on chicken also being found in the former group compared to its more privileged counterpart. Parallel differences in the energy share of these items in the total available food were, however, only subtle, and more evident among food-insecure households than among those living below the poverty line. Our group has previously shown that shares in the total dietary energy were higher for poultry among Brazilians in vulnerable situations, whereas lower for red meat among food-insecure, but not for those with low-income (<0.5 minimum wage per capita) [13]. Consistent with these findings, analyses of inequality in meat consumption in Brazil estimate that individuals earning less than half a minimum wage spend up to 17 times more of their disposable income to purchase one kilogram of red meat compared to those earning more than four minimum wages, despite consuming fattier cuts that are, on average, 32% cheaper [28]. Jointly, these data suggest that strategies adopted by vulnerable Brazilian families to reduce diet costs might be primarily based on diversifying meats rather than simply excluding their consumption. This is reasonably aligned with the recognized cultural values and symbolism of social class associated with meats, particularly red meats, within the Brazilian historical and anthropological context [28,29].

Indeed, more than 60% of Brazilians consume beef and lamb above the EAT-Lancet recommendations [29]. Higher income and male gender are positively associated with this excessive intake, whereas older age and household food insecurity show the opposite effect [29]. Consistently, our results indicate that households in food security and above the poverty line have higher shares in total energy and expenditures from red meat, respectively, while also showing a lower proportion of residents under 18 years of age compared to their respective vulnerable counterparts. Interestingly, households below the food poverty line did not show a clear reduction in red meat availability with declining income, whereas they had a higher frequency of male residents. In turn, a lower energy share from dairy products was observed among food-insecure households. This aligns with previous findings from our group, which associated lower adherence to the EAT-Lancet diet with consumption of dairy products above the recommended level among Brazilians experiencing food security and earning more than two minimum wages, but not among those with severe food insecurity or in the lowest-income class [13]. Therefore, it is suggested that, in addition to financial constraints, low purchasing power, and high food prices, specific demographic characteristics and impaired physical access to meat and dairy products among food-insecure populations also act as barriers to their excessive consumption in Brazil. Since consumption of animal-sourced foods tends to increase with income and food security level, reinforced by local cultural and social norms [28,29], a concerning challenge arises for Brazilian public policies on sustainable food environments, as improving access is required for all components of the EAT-Lancet diet, not only the adequacy ones. In this sense, it is imperative to further explore the future impacts of a recently approved federal decree aiming at exempting taxes on fundamental dietary items, including fresh and minimally processed legumes, cereals, roots, vegetables, and fruits, but also meats and dairy products in the Brazilian basic food basket [30]. Notwithstanding, nutritional requirements of the vulnerable populations, especially women and children, should remain central to discussions on sustainability within the framework of EAT-Lancet diet recommendations, particularly regarding acceptable ranges for consumption of micronutrient-dense, animal-sourced foods [31].

Still comparing budget compositions, we found that, for households in vulnerable situations, relative to more privileged counterparts, shares of both food expenditure and energy availability were higher for refined cereals while lower for all EAT-Lancet diet adequacy components, except legumes. This component accounted for a larger share of expenditures in vulnerable households, despite contributing similarly to energy availability across all assessed strata. In fact, data from the Brazilian Institute of Geography and Statistics (IBGE) indicate that the acquisition and consumption of staple Brazilian cereals and legumes, especially rice and beans, are higher in food-insecure and low-income households [6,17,22]. However, studies analyzing trends among adults from federal capitals have warned of a recent decline in the consumption of these traditional foods across all gender and age groups. Evidence indicates that regular consumption of beans (≥5 times/week) decreased by about 8% between 2006 and 2009 [32], and by about 12% between 2007–2011 and 2012–2017 [33], with projections suggesting that this may no longer be a predominant habit in the country by 2025 [33]. In turn, rice, despite contributing as the major single source of energy in the Brazilian diet (approximately 11%), is consumed predominantly (over 96%) in its processed, refined form [22]. In this regard, the EAT-Lancet diet recommends >32% of calories from whole cereals, but sets no explicit limits for refined grains [1], despite recognizing their adverse health effects [1,34]. Therefore, adapting the Brazilian diet to EAT-Lancet recommendations require efforts not only to preserve regional food patterns, but also to promote a substantial increase in whole grain consumption, irrespective of individuals’ socioeconomic status. For that, cost-reduction measures for staple foods are necessary, as these have demonstrated positive impacts on food security of the poorest populations [35].

Global projection of the most affordable EAT-Lancet diet highlight fruits and vegetables as the largest share of food costs (31.2%), while covering around 8% of total daily energy [2]. In the available food for home consumption in Brazil, the per capita energy contribution of these joint components reached approximately 60% of that recommended level in more privileged households, but less than 50% among those in vulnerable situations, despite accounting for around 15% and 10% of their total food expenditures, respectively. This is unsurprising, given fruits and vegetables are the two most expensive plant-based components of the EAT-Lancet diet in the country [15]. Although Brazil is the third largest fruit producer in the world [36], urgent shifts are needed to align demand with consumption of a variety of vegetables, which are mainly originated from smallholders and family farmers [37]. Also, social inequalities in food deserts and swamps distribution might be related to these findings [38]. Like other fresh foods, fruits and vegetables are typically obtained in open-air markets, greengrocers, from small producers, or street vendors, especially by households experiencing moderate or severe food insecurity [39]. That posit small-scale establishments as strategic strengthening points for public policies aiming to promote healthy diets in Brazil [39]. However, to support the recommendations for increasing consumption of fruit and vegetables, all stages of food systems must be considered. This includes articulating small vendors and street fairs to local family farmers and wholesale distribution centers of fruits and vegetables, especially in underserved regions [37,39], encouraging household-level food production, implementing fiscal policies to reduce prices, and investing in nutrition education [40], beyond protecting low-income families.

An interesting observation was that, while a greater share of total energy availability from refined cereals in food-insecure households was compensated by slightly lower contributions from red meat and dairy products, compared to their counterparts, this shift occurred primarily at the expense of vegetable oils and added sugars among households below the poverty line. In such households, these two EAT-Lancet diet components exhibited energy shares at least 20% and 12% lower, respectively, than those in all other studied situations. Beyond their role as culinary ingredients used in home-cooked meals [11], it is worth noting that vegetable oils and added sugars are also major constituents of ultra-processed foods [41], which might be less frequently consumed among low-income households in the country. In fact, a systematic literature review confirms that, through 2022, contrasting with trends observed in the United States, national surveys from upper-middle-income countries, including Colombia, Mexico, and Brazil, reported a positive association between income and ultra-processed food consumption [42]. Even so, national studies evaluating the impact of cash transfers through Brazil’s largest immediate poverty relief initiative—the Bolsa Família program—suggest that, between 2008 and 2009, beneficiary households increased their per capita total food and energy availability primarily through greater acquisition of vegetable oils, sugar, and other culinary ingredients, as well as fresh and minimally processed meats, potatoes, roots, and vegetables [43], but not necessarily on unhealthy products, such as packaged food [44]. While encouraging, these findings reflect a period in Brazil when ultra-processed products were more expensive than in natura or minimally processed foods, a pattern projected to reverse by 2026 [45]. This poses an additional challenge to promote healthy and sustainable diets in our setting, as an inverse association has been observed between the consumption of these products and adherence to EAT-Lancet diet recommendations [46]. On the other hand, promising debates have recently emerged in Brazil regarding food regulation and marketing policies, particularly concerning the taxation of soft drinks, ready-to-drink teas, and packaged juices [47], and the implementation of front-of-package warnings for products high in sodium, added sugars, and saturated fat [48].

Although the EAT-Lancet reference diet serves as a global benchmark for healthy and sustainable eating, the Brazil’s national dietary guidelines are conceptually aligned with many of its core recommendations, such as moderating the intake of meat and ultra-processed products, and emphasizing fresh and minimally processed foods derived from fair production and distribution practices [11]. Consistently, previous studies have reported that the environmental impact of current Brazilian diets is nearly twice as high as those aligned with both the national guidelines and the EAT-Lancet reference [14,49]. This occurs even with evidence of an inverse correlation between carbon and water footprints and total food costs within each of these dietary patterns [14]. These findings are relevant because they shed light on the economic and environmental mutual benefits of shifting dietary habits in directions suggested by our results and corroborated by the national dietary recommendations, such as replacing excessive meat with more sustainable whole cereals, legumes, nuts, peanuts, fruits, and vegetables [11,14]. Early implementation of these affordable and feasible food choices may help further protect vulnerable populations by contributing to climate change mitigation. This is particularly important as climate change exacerbates food insecurity, especially in low- and middle-income countries, due to its dramatic and socially unequal impacts on local food production, availability, and prices in the face of temperature variations and extreme weather events such as droughts and floods [10,37,50]. Nevertheless, drastic and immediate changes in dietary patterns remain complex and challenging. In this context, the national dietary guidelines—due to their less stringent thresholds and cutoffs proposed for fruits, vegetables, red meat, and discretionary foods, their stronger cultural alignment with local eating habits, and their demonstrated cost-effectiveness and climate benefits [11,14]—may represent a strategic starting point for promoting broader adherence to the EAT-Lancet dietary recommendations in the country.

Some limitations of the present study must be considered. The food security measurement scale used (EBIA) reflects a self-perceived, single dimension of the impaired physical and economic access to foods, without necessarily accounting for dynamics of family food consumption, cultivation, and exchange practices, or long-term stability. Even so, the EBIA is the official instrument validated for studying food insecurity in Brazil [6]. In turn, due to the lack of an official government methodology for measuring poverty, we adopted the World Bank’s proposal specifically calculated using data from the HBS 2017–2018. Although this may hinder comparisons with studies using different administrative or international poverty lines, this criterion was useful in allowing us to identify households not only failing to address basic nutritional requirements (food poverty), but also other non-food necessities (total poverty) [21]. Additionally, we relied on data from household budgets expressed as averages per capita, which may imply four key limitations: (1) short recording period; (2) assumed a uniform distribution of income and expenditures among household members; (3) unmeasured food waste; and (4) did not include meals consumed outside the home. Part of these limitations were addressed by using household aggregate strata as study units and by interpreting EAT-Lancet components not only in terms of absolute amounts, but mainly on their contributions to the total per capita energy availability. This approach, beyond minimizing the impact of varying nutritional requirements of households with different gender and age compositions, has already been shown to provide results closely approximated from the actual food consumption of Brazilians [51]. Finally, although the household budget data used is the most recent available at the national level, they do not reflect the post-COVID-19 pandemic period, which had profound impacts on food insecurity prevalence, population impoverishment, and food price inflation [5,52]. Between 2020 and 2022, for example, nearly half of food-at-home inflation was driven only by animal-sourced foods [53], while the prices of ultra-processed products declined [52]. These recent trends may have increased the financial burden of traditional meals on food-insecure and low-income households, while contributing to their even lower adherence to healthy and sustainable dietary patterns.

Moreover, our results should be interpreted with caution, as reports from the same dataset indicate that out-of-home food had absolute expenditures about 50% lower among mildly and severely food-insecure relative to food-secure households [6]. In addition, a 60% difference has been described in the average contribution of out-of-home food to total energy intake between individuals earning less than half minimum wage (8.2%) or more than two minimum wages (17.8%) [54]. Items described as most consumed out-of-home by Brazilian adults include alcoholic drinks and ultra-processed foods [54]. In this case, the observed differences in average acquisition of EAT-Lancet components between vulnerable and more privileged households might be even more contrasting for added sugars (soft drinks and candies) and vegetable oils (fried snacks), while less marked for refined cereals (pizza, baked snacks, and sandwiches). Also, we applied item-specific correction factors to mitigate the impact of available but uneaten food (e.g., inedible parts and waste during storage and preparation) on our results [26]. Nevertheless, unequal mismatches between per capita food acquisition and dietary intake of EAT-Lancet diet components across the studied household strata may occur, as food waste in Brazil has been shown to vary with consumers’ income levels, though not in a linear manner [55,56]. Finally, it is important take in mind that our analysis primarily focused on describing the composition of food expenditures and availability. However, lower absolute per capita amounts of food and energy were observed among food-insecure and poorer households. On the one hand, this may limit discussions regarding the specific impacts of individual EAT-Lancet components inadequacies. On the other hand, we could predict even worse health effects of unhealthy and unsustainable diets in scenarios of scarcity, since paradoxical associations between lower energy intake and severe food insecurity with both low stature, underweight, and obesity has been described in Brazil, especially among women, with underlying mechanisms and moderating factors yet to be elucidated [57,58].

## 5. Conclusions

Despite allocating substantially different absolute amounts and proportions of their disposable incomes to food, with only minor differences in expenditure composition, households in food insecurity and poverty situations exhibited distinct shares of EAT-Lancet diet components in the energy available for home consumption in Brazil. Compared to their more privileged counterparts, vulnerable households showed a higher reliance on staple items of the traditional Brazilian diet, such as refined cereals and legumes, but lower availability of pricier EAT-Lancet adequacy components, including fruits, vegetables, whole grains, and nuts and peanuts. Compensatory reductions in animal-sourced, moderation components were relatively less evident, although chicken appeared to substitute expenditure for other costlier protein alternatives among food-insecure households. Meanwhile, differences in the energy share of red meat and dairy products occurred modestly among food-insecure households while notably pronounced for added sugars and vegetable oils among those below the poverty line. Even so, the overall low availability of EAT-Lancet diet adequacy components and excess of most moderation components were observed across all studied household strata.

As suggested, increased income alone might not be a guarantee of a more sustainable food acquisition pattern. Therefore, public policies targeting sustainable diets in Brazil should synergically address particularities of different deprivation contexts to ensure food security, reduce income inequalities and promote fair and resilient food systems. That demand government efforts towards assisting low-income families, articulating familiar and smallholders’ food production with food environments in underserved regions, implementing fiscal subsides for healthy and staple foods and tax for ultra-processed products, beyond promoting feasible and culturally acceptable dietary recommendations for Brazilians of varying socioeconomic positions.

## Figures and Tables

**Table 1:** Foods, beverages and ingredients included in the PHDI components. Brazilian Household Budget Survey (HBS) 2017–2018.

PHDI Components	Food and Beverages
**Adequacy components**	
Nuts and peanuts	Nuts, almonds, peanuts, and seeds
Legumes	Beans, chickpeas, lentils, peas, soy and soy products
Fruits	Fesh fruits, dried fruits, coconut water, juices, nectars and punches
Vegetables	All types of vegetables, except tubers
Whole cereals	Brown rice, whole bread, wheat bran, oatmeal and quinoa
**Optimum components**	
Eggs	Chicken and other poultry eggs
Fish and seafood	Freh or canned fish, seafood, squid, shrimp, and crab
Tubers and potatoes	Potatoes, sweet potatoes, yams, cassava and other types
Dairy	Cow and goat milks, yogurts, and cheeses.
Vegetable oils	Olive oils, margarine, seed oils, palm oils
**Moderation components**	
Red meat	Beef, lamb and pork, including their processed products
Chicken	Chicken and other poultry, including their processed products
Animal fats	Lard, tallow, butter and other dairy fats
Added sugars	Table sugar, honey, and the added sugar to manufactured foods
**Omitted component**	
Refined cereals	Polished rice, white breads, refined grain flours and starches

Adapted from Cacau et al. (2022) [25].

**Table 2:** Sociodemographic characterization of Brazilian household strata. Brazilian Household Budget Survey (HBS) 2017–2018.

Indicators	Mean or %	IC 95%
**Household**		
% rural	22.3	(18.6; 26.0)
% North region	6.8	(4.7; 9.0)
% Northeast region	27.7	(23.5; 31.9)
% Southeast region	41.6	(36.0; 47.1)
% South region	15.9	(12.6; 19.3)
% Midwest region	7.9	(5.4; 10.5)
Number of residents	3.0	(3.0; 3.1)
**Household head**		
Average age	50.3	(50.0; 50.6)
% female	41.5	(40.5; 42.5)
% non-white	56.3	(54.1; 58.5)
Years of schooling	7.9	(7.6; 8.0)
**Residents**		
Average age	35.6	(35.2; 36.0)
% female	51.3	(51.0; 51.7)
% non-white	55.2	(53.1; 57.3)
% under 18 years old	25.1	(24.6; 25.7)
Years of schooling (individuals > 18 years old)	9.1	(8.8; 9.2)
Disposable income (USD/capita/day)	19.3	(18.0; 20.6)
**Food security status**		
% food-insecure households	37.9	(36.3; 39.5)
% mild food insecurity households	24.5	(23.6; 25.5)
% moderate food insecurity households	8.5	(7.9; 9.1)
% severe food insecurity households	4.9	(4.4; 5.3)
**Poverty status**		
% households below the total poverty line	12.0	(10.8; 13.2)
% households below the food poverty line	4.0	(3.5; 4.5)

**Table 3:** Sociodemographic characterization of Brazilian household strata according to food insecurity and poverty status. Brazilian Household Budget Survey (HBS) 2017–2018.

Indicators	Food Security		Food Insecurity		Above Poverty Line		Below Poverty Line	
	Mean or %	IC 95%	Mean or %	IC 95%	Mean or %	IC 95%	Mean or %	**IC 95%**
**Household**								
% rural	22.0	(18.3–25.7)	22.7	(18.9–26.4)	22.2	(18.5; 25.9)	24.3	(20.2; 28.4)
% North region	6.7	(4.5–8.8)	7.0	(4.8–9.3)	7.0	(4.7; 9.2)	7.1	(4.9; 9.3)
% Northeast region	27.5	(23.3–31.7)	28.2	(23.9–32.4)	27.7	(23.5; 31.8)	30.4	(25.8; 35.0)
% Southeast region	41.4	(35.7–47)	41.7	(36.1–47.2)	41.4	(35.9; 47.0)	40.7	(34.8; 46.6)
% South region	15.9	(12.6–19.3)	15.5	(12.2–18.8)	16.0	(12.6; 19.3)	13.8	(10.3; 17.2)
% Midwest region	8.5	(5.3–11.7)	7.7	(5.3–10.0)	8.0	(5.4; 10.6)	7.9	(5.5; 10.3)
Number of residents	2.8	(2.8–2.9)	3.3	(3.3–3.3)	2.9	(2.9; 2.9)	4.0	(3.9; 4.1)
**Household head**								
Average age	51.7	(51.3–52.1)	48.2	(47.8–48.6)	51.5	(51.1; 51.9)	42.0	(41.2; 42.8)
% female	38.1	(36.9–39.4)	47.0	(45.5–48.6)	41.3	(40.3; 42.3)	42.3	(39.0; 45.6)
% non-white	53.0	(50.7–55.3)	63.1	(60.9–65.4)	55.6	(53.4; 57.8)	68.7	(65.3; 72.1)
Years of schooling	8.3	(8.1–8.6)	6.9	(6.8–7.1)	8.0	(7.8; 8.2)	5.9	(5.6; 6.2)
**Residents**								
Average age	38.1	(37.7–38.5)	32.3	(31.9–32.8)	37.5	(37.1; 37.8)	26.2	(25.6; 26.9)
% female	51.1	(50.6–51.6)	51.6	(51.1–52.2)	51.3	(50.9; 51.6)	51.2	(49.8; 52.7)
% non-white	51.7	(49.5–53.8)	61.3	(59.2–63.5)	54.3	(52.2; 56.4)	66.7	(63.8; 69.7)
% under 18 years old	21.3	(20.8–21.8)	30.1	(29.4–30.9)	22.4	(21.9; 22.9)	39.4	(38.0; 40.7)
Years of schooling (individuals > 18 years old)	9.5	(9.2–9.7)	8.3	(8.1–8.5)	9.1	(8.9; 9.3)	7.7	(7.5; 7.9)
Disposable income (USD/capita/day)	22.9	(21.4; 24.4)	12.2	(11.6; 12.9)	20.7	(19.4; 21.9)	3.2	(3.1; 3.3)

**Table 4:** EAT-Lancet Diet components acquisition by Brazilian household strata according to food insecurity and poverty status. Brazilian Household Budget Survey (HBS) 2017–2018.

Food Components		Food Security		Food Insecurity		Above Poverty Line		Below Poverty Line	
		Mean	IC 95%	Mean	IC 95%	Mean	IC 95%	Mean	IC 95%
Total food	Amount (g/capita/day)	664.4	(645.0; 683.8)	513.2	(495.6; 530.8)	642.6	(626.6; 658.7)	413.8	(358.6; 468.9)
	Energy (kcal/capita/day)	1307.0	(1270.4; 1343.6)	1128.8	(1085.3; 1172.3)	1303.5	(1270.5; 1336.6)	917.8	(814.0; 1021.5)
	Expenditure (USD/capita/day)	1.5	(1.4; 1.5)	1.0	(1.0; 1.0)	1.4	(1.3; 1.4)	0.8	(0.6; 0.9)
Nuts and peanuts	Amount (g/capita/day)	1.6	(1.3; 1.9)	0.8	(0.6; 0.9)	1.5	(1.2; 1.7)	0.3	(0.2; 0.4)
	Energy share (%/capita/day)	0.3	(0.2; 0.4)	0.2	(0.1; 0.2)	0.3	(0.2; 0.4)	0.1	(0.0; 0.1)
	Expenditure share (%/capita/day)	0.5	(0.4; 0.6)	0.3	(0.2; 0.4)	0.5	(0.4; 0.5)	0.1	(0.1; 0.2)
Legumes	Amount (g/capita/day)	19.1	(17.9; 20.4)	18.8	(17.3; 20.2)	20.0	(18.8; 21.1)	21.2	(11.9; 30.6)
	Energy share (%/capita/day)	4.2	(4.0; 4.5)	4.7	(4.4; 5.0)	4.4	(4.2; 4.6)	4.6	(3.9; 5.4)
	Expenditure share (%/capita/day)	1.8	(1.7; 2.0)	2.3	(2.2; 2.5)	2.0	(1.9; 2.1)	2.4	(2.1; 2.8)
Fruits	Amount (g/capita/day)	103.1	(97.2; 108.9)	56.2	(52.5; 60.0)	92.2	(87.6; 96.8)	42.5	(29.7; 55.3)
	Energy share (%/capita/day)	4.3	(4.1; 4.5)	2.9	(2.7; 3.0)	3.9	(3.7; 4.0)	2.8	(2.1; 3.6)
	Expenditure share (%/capita/day)	7.8	(7.5; 8.1)	5.9	(5.6; 6.2)	7.4	(7.1; 7.7)	5.0	(3.8; 6.1)
Vegetables	Amount (g/capita/day)	58.0	(55.2; 60.8)	38.0	(35.8; 40.2)	53.7	(51.4; 56.1)	32.5	(21.2; 43.8)
	Energy share (%/capita/day)	1.3	(1.2; 1.4)	1.0	(1.0; 1.1)	1.2	(1.2; 1.3)	1.0	(0.9; 1.2)
	Expenditure share (%/capita/day)	6.6	(6.4; 6.9)	6.0	(5.7; 6.3)	6.5	(6.3; 6.7)	5.4	(4.9; 5.9)
Whole cereals	Amount (g/capita/day)	13.7	(12.4; 14.9)	8.1	(7.3; 9.0)	13.0	(12.0; 14.0)	5.5	(4.4; 6.7)
	Energy share (%/capita/day)	1.1	(1.0; 1.2)	0.7	(0.6; 0.8)	1.0	(0.9; 1.1)	0.6	(0.5; 0.8)
	Expenditure share (%/capita/day)	1.1	(1.1; 1.2)	0.8	(0.8; 0.9)	1.1	(1.0; 1.2)	0.7	(0.5; 0.8)
Refined cereals	Amount (g/capita/day)	430.9	(416.6; 445.2)	418.4	(399.7; 437.2)	444.1	(430.0; 458.1)	347.2	(311.5; 383.0)
	Energy share (%/capita/day)	33.0	(32.3; 33.6)	37.0	(36.2; 37.7)	34.0	(33.4; 34.6)	37.2	(35.1; 39.3)
	Expenditure share (%/capita/day)	19.9	(19.4; 20.3)	23.1	(22.5; 23.7)	20.6	(20.2; 21.0)	25.1	(23.1; 27.0)
Eggs	Amount (g/capita/day)	11.1	(10.3; 11.8)	8.3	(7.7; 8.9)	10.7	(10.1; 11.4)	6.7	(4.9; 8.5)
	Energy share (%/capita/day)	1.0	(0.9; 1.1)	0.9	(0.8; 1.0)	1.0	(0.9; 1.0)	1.0	(0.8; 1.3)
	Expenditure share (%/capita/day)	2.0	(1.9; 2.2)	2.1	(2.0; 2.3)	2.0	(1.9; 2.2)	2.3	(1.9; 2.7)
Fish and shellfish	Amount (g/capita/day)	8.5	(7.4; 9.7)	6.5	(5.5; 7.5)	8.6	(7.4; 9.8)	5.1	(3.5; 6.8)
	Energy share (%/capita/day)	0.7	(0.6; 0.7)	0.6	(0.5; 0.7)	0.6	(0.6; 0.7)	0.5	(0.4; 0.6)
	Expenditure share (%/capita/day)	2.8	(2.5; 3.1)	2.5	(2.2; 2.8)	2.7	(2.4; 3.0)	2.0	(1.6; 2.4)
Potatoes and tubers	Amount (g/capita/day)	36.6	(34.6; 38.7)	27.9	(26.2; 29.6)	35.6	(33.8; 37.4)	19.1	(16.0; 22.2)
	Energy share (%/capita/day)	4.2	(3.9; 4.5)	3.9	(3.5; 4.2)	4.1	(3.8; 4.4)	4.1	(3.4; 4.7)
	Expenditure share (%/capita/day)	3.4	(3.3; 3.6)	3.4	(3.3; 3.6)	3.4	(3.3; 3.6)	3.1	(2.7; 3.4)
Dairy products	Amount (g/capita/day)	104.4	(99.0; 109.8)	76.9	(71.3; 82.4)	98.2	(93.5; 102.9)	63.1	(53.0; 73.3)
	Energy share (%/capita/day)	8.1	(7.8; 8.4)	6.7	(6.4; 7.1)	7.6	(7.3; 7.9)	7.5	(6.5; 8.6)
	Expenditure share (%/capita/day)	10.4	(10.1; 10.7)	9.6	(9.1; 10.0)	10.1	(9.9; 10.4)	9.8	(8.8; 10.9)
Vegetable oils	Amount (g/capita/day)	23.1	(20.7; 25.5)	20.2	(18.6; 21.7)	23.3	(21.4; 25.2)	14.9	(12.8; 17.1)
	Energy share (%/capita/day)	13.4	(12.8; 14.0)	14.0	(13.4; 14.7)	13.8	(13.3; 14.4)	11.2	(10.2; 12.3)
	Expenditure share (%/capita/day)	2.8	(2.7; 3.0)	3.3	(3.1; 3.5)	3.0	(2.9; 3.1)	3.1	(2.8; 3.5)
Red meat	Amount (g/capita/day)	69.8	(67.5; 72.2)	51.7	(49.2; 54.3)	66.9	(64.9; 69.0)	39.7	(33.2; 46.2)
	Energy share (%/capita/day)	9.5	(9.2; 9.7)	8.7	(8.3; 9.1)	9.2	(8.9; 9.4)	8.7	(7.7; 9.7)
	Expenditure share (%/capita/day)	23.2	(22.6; 23.9)	22.3	(21.5; 23.1)	23.0	(22.5; 23.5)	20.0	(18.6; 21.4)
Chicken	Amount (g/capita/day)	39.3	(37.4; 41.2)	34.1	(32.1; 36.1)	39.4	(37.6; 41.2)	26.2	(21.9; 30.5)
	Energy share (%/capita/day)	3.8	(3.6; 3.9)	3.8	(3.6; 4.0)	3.7	(3.6; 3.8)	3.9	(3.3; 4.4)
	Expenditure share (%/capita/day)	7.5	(7.1; 7.8)	8.8	(8.3; 9.2)	7.8	(7.5; 8.1)	8.5	(7.7; 9.3)
Animal fat	Amount (g/capita/day)	2.9	(2.7; 3.2)	1.7	(1.5; 1.9)	2.6	(2.4; 2.7)	0.9	(0.7; 1.1)
	Energy share (%/capita/day)	1.0	(0.9; 1.2)	0.7	(0.6; 0.8)	0.9	(0.8; 1.0)	0.5	(0.4; 0.7)
	Expenditure share (%/capita/day)	1.0	(0.9; 1.1)	0.7	(0.6; 0.8)	0.9	(0.8; 1.0)	0.5	(0.4; 0.6)
Added sugar	Amount (g/capita/day)	47.6	(45.2; 50.0)	42.1	(39.7; 44.4)	47.6	(45.8; 49.4)	34.7	(28.5; 40.9)
	Energy share (%/capita/day)	14.2	(13.7; 14.7)	14.3	(13.9; 14.8)	14.3	(13.9; 14.6)	12.6	(11.5; 13.7)
	Expenditure share (%/capita/day)	9.0	(8.7; 9.4)	8.8	(8.4; 9.3)	9.0	(8.7; 9.3)	8.3	(7.4; 9.2)

## Data Availability

This study analyzed publicly available datasets. These data can be accessed at https://www.ibge.gov.br/estatisticas/sociais/populacao/24786-pesquisa-de-orcamentosfamiliares-2.html?=&t=microdados (accessed on 30 March 2025).

## Supplementary Materials

The following supporting information can be downloaded at: https://www.mdpi.com/article/10.3390/ijerph22050808/s1, Table S1: Sociodemographic characterization of Brazilian household strata according levels food insecurity and poverty. Brazilian Household Budget Survey (HBS) 2017–2018; Table S2: EAT-Lancet Diet components acquisition by Brazilian household strata according to levels of food insecurity and poverty. Brazilian Household Budget Survey (HBS) 2017–2018.

## Abbreviations

The following abbreviations are used in this manuscript:

HBS	Household Budget Survey
EBIA	Brazilian Food Insecurity Scale
IBGE	Brazilian Institute of Geography and Statistics
TBCA	Brazilian Food Composition Table
PHDI	Planetary Health Diet Index
