# Supplementary material for: EAT-Lancet Diet Components Acquisition According to Food Insecurity and Poverty Status in Brazil: An Analysis of National Household Budget Survey 2017–2018
Source: Int J Environ Res Public Health. 2025 May 21;22(5):808. doi: 10.3390/ijerph22050808 (PMC12110852; doi:10.3390/ijerph22050808)
Supplement: Supplementary file 1 [file ijerph-22-00808-s001.zip › ijerph-3590117-supplementary.pdf]

Table S1. Sociodemographic characterization of Brazilian household strata according levels food insecurity and poverty. Brazilian Household Budget Survey (HBS) 2017-2018.

Indicators	Food Security		Mild or moderate food insecurity		Severe food insecurity		Above total poverty line		Between poverty lines		Below food poverty line	
	**Mean or %**	**IC 95%**	**Mean or %**	**IC 95%**	**Mean or %**	**IC 95%**	**Mean or %**	**IC 95%**	**Mean or %**	**IC 95%**	**Mean or %**	**IC 95%**
**Household**												
% rural	22.0	(18.3 - 25.7)	22.8	(19.0 - 26.6)	22.4	(18.4 - 26.4)	22.2	(18.5; 25.9)	25.0	(20.7; 29.3)	26.1	(21.2; 31.0)
% North region	6.7	(4.5 - 8.8)	7.1	(4.8 - 9.3)	7.7	(5.2 - 10.1)	7.0	(4.7; 9.2)	7.4	(5.1; 9.8)	9.6	(6.7; 12.4)
% Northeast region	27.5	(23.3 - 31.7)	28.3	(24.0 - 32.6)	31.0	(26.3 - 35.7)	27.7	(23.5; 31.8)	31.8	(27.0; 36.6)	37.6	(31.8; 43.3)
% Southeast region	41.4	(35.7 - 47.0)	41.7	(36.2 - 47.3)	38.3	(32.3 - 44.3)	41.4	(35.9; 47.0)	39.6	(33.6; 45.6)	37.2	(30.2; 44.1)
% South region	15.9	(12.6 - 19.3)	15.3	(12.0 - 18.6)	14.1	(10.4 - 17.8)	16.0	(12.6; 19.3)	13.6	(10.1; 17.0)	9.4	(6.1; 12.8)
% Midwest region	8.5	(5.4 - 11.7)	7.6	(5.3 - 9.9)	8.9	(6.1 - 11.8)	8.0	(5.4; 10.6)	7.6	(5.2; 10.0)	6.2	(3.8; 8.6)
Number of residents	2.8	(2.8 - 2.9)	3.3	(3.3 - 3.4)	3.1	(3.0 - 3.2)	2.9	(2.9; 2.9)	4.1	(4.0; 4.2)	3.8	(3.7; 4.0)
**Household head**												
Average age	51.7	(51.3 - 52.1)	48.1	(47.7 - 48.6)	48.5	(47.6 - 49.5)	51.5	(51.1; 51.9)	42.2	(41.3; 43.1)	41.8	(40.4; 43.1)
% female	38.1	(36.9 - 39.4)	46.6	(45.0 - 48.2)	53.0	(49.3 - 56.7)	41.3	(40.3; 42.3)	44.3	(40.7; 47.8)	39.5	(35.1; 43.9)
% non-white	53.0	(50.7 - 55.3)	62.9	(60.6 - 65.1)	68.7	(64.7 - 72.7)	55.6	(53.4; 57.8)	68.8	(65.3; 72.2)	72.8	(68.6; 77.1)
Years of schooling	8.3	(8.1 - 8.6)	7.0	(6.8 - 7.2)	6.2	(5.9 - 6.5)	8.0	(7.8; 8.2)	5.8	(5.5; 6.1)	6.1	(5.6; 6.5)
**Residents**												
Average age	38.1	(37.7 - 38.5)	32.4	(31.9 - 32.8)	34.2	(32.9 - 35.5)	37.5	(37.1; 37.8)	26.1	(25.5; 26.7)	27.1	(25.8; 28.5)
% female	51.1	(50.6 - 51.6)	51.9	(51.2 - 52.5)	51.7	(49.7 - 53.7)	51.3	(50.9; 51.6)	52.1	(50.8; 53.4)	48.5	(46.4; 50.7)
% non-white	51.7	(49.5 - 53.8)	60.9	(58.8 - 63.1)	67.4	(63.7 - 71.1)	54.3	(52.2; 56.4)	67.2	(64.4; 70.1)	70.4	(66.4; 74.5)
% under 18 years old	21.3	(20.8 - 21.8)	29.9	(29.2 - 30.7)	28.5	(26.2 - 30.7)	22.4	(21.9; 22.9)	39.6	(38.4; 40.8)	37.1	(34.4; 39.7)
Years of schooling (> 18 y.o.)	9.5	(9.2 - 9.7)	8.4	(8.2 - 8.6)	7.0	(6.7 - 7.3)	9.1	(8.9; 9.3)	7.6	(7.4; 7.8)	7.6	(7.2; 7.9)
Income (US$/capita/day)	22.9	(21.4; 24.4)	12.5	(11.6; 13.2)	10.7	(9.8; 11.7)	20.7	(19.4; 21.9)	3.8	(3.7; 3.8)	1.6	(1.4; 1.8)

Table S2. EAT-Lancet Diet components acquisition by Brazilian household strata according to levels of food insecurity and poverty. Brazilian Household Budget Survey (HBS) 2017-2018.

	**Food components**	**Food Security**		**Mild or moderate food insecurity**		**Severe food insecurity**		**Above total poverty line**		**Between poverty lines**		**Below food poverty line**	
		**Mean**	**IC 95%**	**Mean**	**IC 95%**	**Mean**	**IC 95%**	**Mean**	**IC 95%**	**Mean**	**IC 95%**	**Mean**	**IC 95%**
**Total food**	Amount (g/capita/day)	664.4	(645.0; 683.8)	523.9	(505.2; 542.7)	449.4	(406.8; 491.9)	642.6	(626.6; 658.7)	395.7	(358.1; 433.3)	440.1	(341.1; 539.0)
	Energy (kcal/capita/day)	1307.0	(1270.4; 1343.5)	1146.6	(1099.7; 1193.6)	1044.5	(931.2; 1157.8)	1303.5	(1270.5; 1336.6)	903.2	(821.3; 985.1)	960.1	(766.1; 1154.1)
	Expenditure (US$/capita/day)	1.5	(1.4; 1.5)	1.0	(1.0; 1.1)	0.8	(0.8; 0.9)	1.4	(1.3; 1.4)	0.7	(0.7; 0.8)	0.9	(0.6; 1.1)
**Nuts and peanuts**	Amount (g/capita/day)	1.6	(1.3; 1.9)	0.8	(0.6; 1.0)	0.6	(0.2; 0.9)	1.5	(1.2; 1.7)	0.3	(0.1; 0.4)	0.4	(0.1; 0.7)
	Energy share (%/capita/day)	0.3	(0.2; 0.4)	0.2	(0.1; 0.2)	0.1	(0.0; 0.2)	0.3	(0.2; 0.4)	0.1	(0.0; 0.1)	0.1	(0.0; 0.1)
	Expenditure share (%/capita/day)	0.5	(0.4; 0.6)	0.3	(0.2; 0.4)	0.2	(0.1; 0.2)	0.5	(0.4; 0.5)	0.1	(0.1; 0.2)	0.1	(0.1; 0.2)
**Legumes**	Amount (g/capita/day)	19.1	(17.9; 20.4)	18.7	(17.2; 20.1)	20.7	(17.0; 24.5)	20.0	(18.8; 21.1)	16.8	(11.1; 22.4)	23.5	(12.2; 34.8)
	Energy share (%/capita/day)	4.2	(4.0; 4.5)	4.6	(4.3; 4.9)	4.4	(3.8; 5.0)	4.4	(4.2; 4.6)	4.2	(3.6; 4.8)	4.3	(3.4; 5.3)
	Expenditure share (%/capita/day)	1.8	(1.7; 2.0)	2.2	(2.1; 2.4)	2.8	(2.2; 3.3)	2.0	(1.9; 2.1)	2.4	(2.0; 2.7)	2.4	(1.9; 2.9)
**Fruits**	Amount (g/capita/day)	103.1	(97.2; 108.9)	58.1	(54.3; 62.0)	40.5	(31.4; 49.5)	92.2	(87.6; 96.8)	34.0	(26.9; 41.2)	55.4	(31.5; 79.2)
	Energy share (%/capita/day)	4.3	(4.1; 4.5)	2.9	(2.7; 3.1)	3.1	(2.3; 4.0)	3.9	(3.7; 4.0)	2.9	(2.1; 3.7)	2.5	(1.8; 3.2)
	Expenditure share (%/capita/day)	7.8	(7.5; 8.1)	5.9	(5.6; 6.3)	5.4	(4.3; 6.5)	7.4	(7.1; 7.7)	5.0	(3.7; 6.2)	4.3	(3.4; 5.1)
**Vegetables**	Amount (g/capita/day)	58.0	(55.2; 60.8)	38.8	(36.6; 41.0)	32.4	(26.1; 38.8)	53.7	(51.4; 56.1)	26.5	(21.6; 31.4)	37.3	(21.3; 53.3)
	Energy share (%/capita/day)	1.3	(1.2; 1.4)	1.1	(1.0; 1.1)	1.2	(0.8; 1.5)	1.2	(1.2; 1.3)	1.0	(0.9; 1.2)	1.6	(0.9; 2.3)
	Expenditure share (%/capita/day)	6.6	(6.4; 6.9)	6.0	(5.7; 6.3)	5.3	(4.6; 6.0)	6.5	(6.3; 6.7)	5.3	(4.7; 5.8)	5.7	(4.6; 6.8)
**Whole cereals**	Amount (g/capita/day)	13.7	(12.4; 14.9)	8.6	(7.7; 9.6)	6.1	(4.5; 7.7)	13.0	(12.0; 14.0)	4.8	(3.6; 5.9)	11.7	(3.8; 19.7)
	Energy share (%/capita/day)	1.1	(1.0; 1.2)	0.7	(0.7; 0.8)	0.7	(0.4; 0.9)	1.0	(0.9; 1.1)	0.6	(0.4; 0.8)	1.0	(0.6; 1.3)
	Expenditure share (%/capita/day)	1.1	(1.1; 1.2)	0.9	(0.8; 0.9)	0.8	(0.5; 1.0)	1.1	(1.0; 1.2)	0.6	(0.4; 0.9)	1.0	(0.6; 1.3)
**Refined cereals**	Amount (g/capita/day)	430.9	(416.6; 445.2)	422.7	(402.0; 443.5)	406.3	(354.6; 457.9)	444.1	(430.0; 458.1)	355.8	(323.0; 388.6)	330.5	(274.0; 387.0)
	Energy share (%/capita/day)	33.0	(32.3; 33.6)	36.5	(35.6; 37.3)	37.1	(34.4; 39.7)	34.0	(33.4; 37.3)	38.1	(35.9; 37.3)	34.7	(32.1; 37.3)
	Expenditure share (%/capita/day)	19.9	(19.4; 20.3)	22.9	(22.3; 23.6)	26.0	(23.6; 28.3)	20.6	(20.2; 21.0)	25.8	(23.7; 27.8)	22.8	(20.7; 24.8)
**Eggs**	Amount (g/capita/day)	11.1	(10.3; 11.8)	8.3	(7.7; 8.9)	9.0	(5.4; 12.6)	10.7	(10.1; 11.4)	5.8	(4.9; 6.7)	6.6	(4.1; 9.1)
	Energy share (%/capita/day)	1.0	(0.9; 1.1)	0.9	(0.8; 0.9)	0.9	(0.7; 1.2)	1.0	(0.9; 1.0)	1.0	(0.7; 1.3)	1.0	(0.7; 1.4)
	Expenditure share (%/capita/day)	2.0	(1.9; 2.2)	2.1	(1.9; 2.2)	2.2	(1.8; 2.7)	2.0	(1.9; 2.2)	2.2	(1.8; 2.7)	2.2	(1.6; 2.7)
	Amount (g/capita/day)	8.5	(7.4; 9.7)	6.6	(5.6; 7.7)	5.9	(4.5; 7.3)	8.6	(7.4; 9.8)	5.8	(4.0; 7.6)	4.8	(3.1; 6.5)

**Fish and shellfish**	Energy share (%/capita/day)	0.7	(0.6; 0.7)	0.6	(0.5; 0.6)	0.7	(0.5; 1.0)	0.6	(0.6; 0.7)	0.5	(0.4; 0.6)	0.5	(0.4; 0.7)
	Expenditure share (%/capita/day)	2.8	(2.5; 3.1)	2.5	(2.2; 2.8)	2.3	(1.7; 2.9)	2.7	(2.5; 3.0)	2.1	(1.7; 2.5)	2.1	(1.6; 2.7)
**Potatoes and tubers**	Amount (g/capita/day)	36.6	(34.6; 38.7)	28.3	(26.5; 30.1)	25.9	(21.3; 30.4)	35.6	(33.8; 37.4)	20.2	(17.0; 23.4)	19.9	(15.3; 24.6)
	Energy share (%/capita/day)	4.2	(3.9; 4.5)	3.8	(3.5; 4.1)	4.4	(3.5; 5.3)	4.1	(3.8; 4.4)	4.0	(3.4; 4.6)	4.4	(3.3; 5.5)
	Expenditure share (%/capita/day)	3.4	(3.3; 3.6)	3.4	(3.2; 3.6)	3.7	(3.0; 4.5)	3.4	(3.3; 3.6)	3.1	(2.7; 3.4)	3.0	(2.3; 3.6)
**Dairy products**	Amount (g/capita/day)	104.4	(99.0; 109.8)	79.8	(73.9; 85.6)	53.7	(44.1; 63.3)	98.2	(93.5; 102.9)	61.7	(50.9; 72.5)	56.8	(38.1; 75.5)
	Energy share (%/capita/day)	8.1	(7.8; 8.4)	6.9	(6.5; 7.3)	6.3	(5.1; 7.6)	7.6	(7.3; 7.9)	7.7	(6.4; 9.1)	6.6	(5.3; 7.9)
	Expenditure share (%/capita/day)	10.4	(10.1; 10.7)	9.7	(9.3; 10.1)	7.7	(6.5; 9.0)	10.1	(9.9; 10.4)	9.8	(8.5; 11.0)	8.7	(7.4; 10.0)
**Vegetable oils**	Amount (g/capita/day)	23.1	(20.7; 25.5)	20.4	(18.8; 22.1)	18.1	(14.8; 21.4)	23.3	(21.4; 25.2)	14.7	(12.6; 16.8)	15.0	(10.8; 19.2)
	Energy share (%/capita/day)	13.4	(12.8; 14.0)	14.0	(13.3; 14.7)	11.0	(9.2; 12.8)	13.8	(13.3; 14.4)	10.9	(9.7; 12.1)	9.2	(7.4; 11.0)
	Expenditure share (%/capita/day)	2.8	(2.7; 3.0)	3.3	(3.1; 3.5)	3.3	(2.5; 4.1)	3.0	(2.9; 3.1)	3.1	(2.7; 3.5)	2.8	(2.1; 3.5)
**Red meat**	Amount (g/capita/day)	69.8	(67.5; 72.2)	52.7	(50.3; 55.2)	49.3	(38.3; 60.3)	66.9	(64.9; 69.0)	39.5	(35.0; 44.0)	44.5	(31.4; 57.6)
	Energy share (%/capita/day)	9.5	(9.2; 9.7)	8.9	(8.4; 9.4)	8.8	(7.4; 10.3)	9.2	(8.9; 9.4)	8.7	(7.7; 9.7)	10.5	(7.7; 13.4)
	Expenditure share (%/capita/day)	23.2	(22.6; 23.9)	22.5	(21.6; 23.3)	18.8	(16.9; 20.8)	23.0	(22.5; 23.5)	20.3	(18.7; 22.0)	19.8	(16.5; 23.1)
**Chicken**	Amount (g/capita/day)	39.3	(37.4; 41.2)	34.8	(32.8; 36.9)	31.0	(26.0; 36.0)	39.4	(37.6; 41.2)	27.8	(23.3; 32.4)	27.0	(19.7; 34.4)
	Energy share (%/capita/day)	3.8	(3.6; 3.9)	3.9	(3.7; 4.1)	4.2	(3.3; 5.1)	3.7	(3.6; 3.8)	3.9	(3.4; 4.4)	3.9	(3.1; 4.7)
	Expenditure share (%/capita/day)	7.5	(7.1; 7.8)	8.7	(8.3; 9.2)	9.1	(7.7; 10.4)	7.8	(7.5; 8.1)	8.4	(7.6; 9.2)	8.7	(7.3; 10.0)
**Animal fat**	Amount (g/capita/day)	2.9	(2.7; 3.2)	1.8	(1.6; 2.0)	1.0	(0.6; 1.4)	2.6	(2.4; 2.7)	0.9	(0.7; 1.1)	1.1	(0.6; 1.7)
	Energy share (%/capita/day)	1.0	(0.9; 1.2)	0.7	(0.6; 0.8)	0.5	(0.2; 0.8)	0.9	(0.8; 1.0)	0.5	(0.3; 0.7)	0.6	(0.2; 1.0)
	Expenditure share (%/capita/day)	1.0	(0.9; 1.1)	0.7	(0.6; 0.8)	0.4	(0.2; 0.6)	0.9	(0.8; 1.0)	0.4	(0.3; 0.6)	0.6	(0.2; 1.1)
**Added sugar**	Amount (g/capita/day)	47.6	(45.2; 50.0)	43.0	(40.5; 45.5)	36.7	(31.0; 42.3)	47.6	(45.8; 49.4)	33.1	(27.7; 38.5)	40.0	(25.4; 54.6)
	Energy share (%/capita/day)	14.2	(13.7; 14.7)	14.4	(13.9; 14.9)	11.8	(10.5; 13.1)	14.3	(13.9; 14.6)	12.0	(10.8; 13.1)	11.9	(10.4; 13.4)
	Expenditure share (%/capita/day)	9.0	(8.7; 9.4)	8.9	(8.4; 9.4)	7.2	(6.2; 8.2)	9.0	(8.7; 9.3)	7.5	(6.7; 8.3)	8.6	(7.0; 10.3)
